# Piecewise exponential models to assess the influence of job-specific experience on the hazard of acute injury for hourly factory workers

**DOI:** 10.1186/1471-2288-13-89

**Published:** 2013-07-10

**Authors:** Jessica Kubo, Mark R Cullen, Linda Cantley, Martin Slade, Baylah Tessier-Sherman, Oyebode Taiwo, Manisha Desai

**Affiliations:** 1 Quantitative Sciences Unit, Stanford University, Palo Alto, CA, USA; 2 Department of Medicine, Stanford University, Stanford, CA, USA; 3 Department of Internal Medicine, Yale University, New Haven, CT, USA

**Keywords:** Piecewise exponential models, Weibull models, Baseline hazard, Time to event data, Occupational health, Censored data, Frailty models, Survival analysis

## Abstract

**Background:**

An inverse relationship between experience and risk of injury has been observed in many occupations. Due to statistical challenges, however, it has been difficult to characterize the role of experience on the hazard of injury. In particular, because the time observed up to injury is equivalent to the amount of experience accumulated, the baseline hazard of injury becomes the main parameter of interest, excluding Cox proportional hazards models as applicable methods for consideration.

**Methods:**

Using a data set of 81,301 hourly production workers of a global aluminum company at 207 US facilities, we compared competing parametric models for the baseline hazard to assess whether experience affected the hazard of injury at hire and after later job changes. Specific models considered included the exponential, Weibull, and two (a hypothesis-driven and a data-driven) two-piece exponential models to formally test the null hypothesis that experience does not impact the hazard of injury.

**Results:**

We highlighted the advantages of our comparative approach and the interpretability of our selected model: a two-piece exponential model that allowed the baseline hazard of injury to change with experience. Our findings suggested a 30% increase in the hazard in the first year after job initiation and/or change.

**Conclusions:**

Piecewise exponential models may be particularly useful in modeling risk of injury as a function of experience and have the additional benefit of interpretability over other similarly flexible models.

## Background

There is abundant evidence that traumatic injuries occur more commonly in newly hired workers, suggesting that inexperience may be a risk factor for occupational injury. The relationship between experience and risk of injury has been evaluated in several occupations including aluminum manufacturing [1,2], farming [3], nursing [4,5], steel manufacturing [6], railway work [7] and through analysis of larger injury databases [8,9]. There are several methodological challenges, however, that can greatly influence interpretation of results examining this association. A common approach to addressing this question has been to compare rates of injury between newly hired employees and longer-term employees by making use of cross-sectional case–control studies [3-5,7,9-13]. The potential for confounding, however, is great. New hires are unlikely to be representative of workers in general. In addition to being younger, various “negative” characteristics, such as personal instability, poor work attendance, lesser health, and poor work ethic among others are likely to be overrepresented in this pool. Moreover, “first jobs” are also not representative of all jobs; it is commonplace for newer hires to assume the least desirable of the tasks, and “graduate” into safer and more palatable jobs over time.

Importantly, a cross-sectional assessment does not provide the newly hired employee an opportunity to be observed as an experienced employee or vice versa, allowing each employee to serve as his/her own control. Longitudinal studies are equipped for this purpose and many have made use of such designs [6,8,9,14-16]. These studies, however, did not exploit the longitudinal nature of the data, but rather dealt with it as a nuisance [6], if at all. An additional challenge occurs when employees are followed for varying lengths of time, which affects the probability of observing injury during the period studied. Finally, none of the studies have addressed the complication encountered when employees change jobs within the same industry, often with exposure to new and different tasks.

The overall goal of this study is to assess whether experience affects the risk of acute injury in a population of hourly factory workers of a global aluminum company by paying close attention to the statistical issues raised above. For example, the length of time observed until injury needs to be considered as well as whether the employee was injured while observed. As individuals may or may not be injured during their period of study, employees may be censored, bringing to mind survival analytic tools to handle such data. A complication, however, is that the length of time to injury also defines the level of experience, making standard semi-parametric models like Cox proportional hazards (Cox PH) models inapplicable. To that end, we argue that the parameter of interest is the baseline hazard of injury itself. In this paper, we demonstrate the use of comparing competing parametric survival models for the baseline hazard when it is the main parameter of interest. In particular we illustrate the use of piecewise exponential models that enable changes in the baseline hazard in order to evaluate the influence of experience on the hazard of injury [17]. While we are not the first to utilize such models [18,19], this is a novel application of these methods.

## Methods

**Figure 1:**
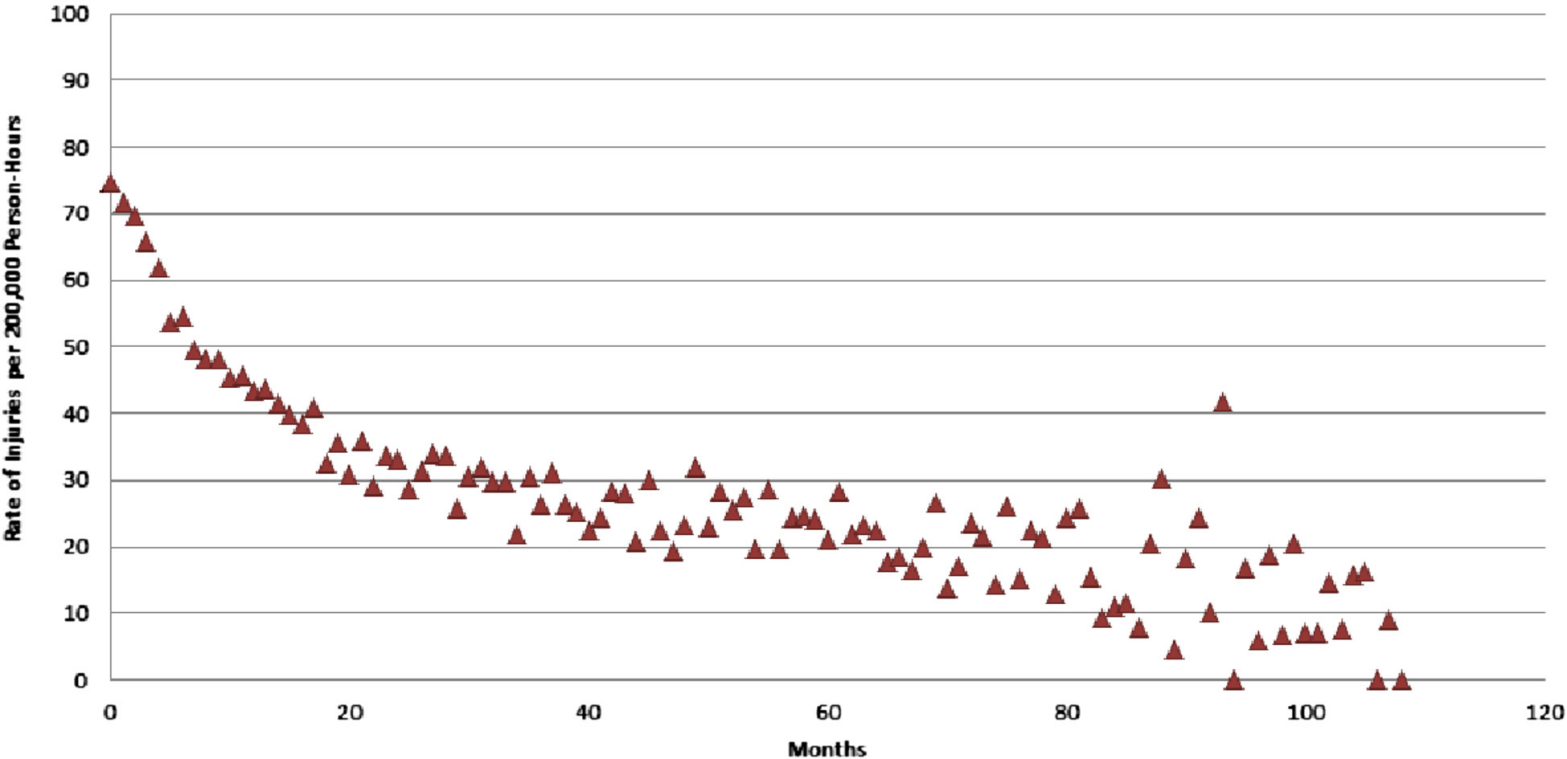
Rates of injury per 200 000 person-hours.

The systematic determinants of injury in manufacturing have been an area of substantial investigation and public health concern. Previous studies by our group in the aluminum industry have already demonstrated several important risks including female gender, lower age, high physical demand, long work hours, obesity and low worker engagement [1,2,20,21]. Crude data (see Figure 1, below) have highlighted concern about the relationship between job experience and risk.

### Study population

Our study population included 81,301 hourly production workers of a global aluminum company at 207 US facilities (90 with >250 employees) employed during the time period spanning January 1, 1996 through December 31, 2007. Data were obtained from a variety of administrative sources including a real time incident management system capturing every injury since 1989, and the electronic human resources data. These datasets are described in greater detail in previous publications [1,2,21]. Notably, there is no reliance on any self-report or survey data.

### Definition of acute injury

Acute injuries included events such as chemical/thermal burns, bruises, cuts/abrasions, fractures, instantaneous musculoskeletal injuries, and other acute incidents. Chronic conditions or events that were cumulative in nature, classified as “near-misses” or not directly related to work did not meet the criteria for acute injury.

### Statistical methods

#### Primary outcome

The primary outcome was time in months from the start of a particular job to injury on that job, where multiple injuries may occur. Non-injured employees were censored at the time corresponding to the earliest occurrence of the following: change of job within the corporation, death, termination from the company, or the end of the observation period, December 31, 2007. Individuals with multiple jobs (ie, who changed jobs during the observation period) and/or multiple injuries on a particular job had multiple outcomes reflecting the length of time from the start of a particular job to observed injury on that job. Once a job change was initiated, the employee was newly considered inexperienced, where a job change involved a significant change in job title. In sensitivity analyses, we considered restricting injuries to those of greater severity.

### Potential confounders

We considered the following important confounders: age and overall company tenure at job initiation, gender, race, physical demand of the job, union status of the plant, plant type (e.g., whether it included a smelting facility), and socio-demographic characteristics.

#### Comparison of competing models for investigating research question

To evaluate the association between the hazard of injury and experience, we modeled the hazard of injury as a function of time on the job (i.e., experience) and other relevant confounders using the Andersen-Gill approach for incorporating recurrent events and time-varying covariates [22,23]. Cox PH models were not applicable for our purposes, as our main parameter of interest was the baseline hazard itself; assessing whether the hazard for injury changes with experience was equivalent to addressing whether the baseline hazard of injury, *λ*_*0*_*(t)*, was constant over the time on the job (i.e., whether *λ*_*0*_*(t)=λ*_*0*_, the form of an exponential baseline hazard).

Our approach to addressing the research question, therefore, was to compare competing baseline hazards. As described above, a parametric model that assumes a constant baseline hazard is the exponential model, where *λ*_*i*_*(t,x*_*i*_*)=λ*_*0*_exp(*x’*_*i*_*β*). We assumed this model under the null hypothesis (i.e., that experience does not affect the hazard of injury).

Three models are considered under the alternative hypothesis: the Weibull and two piecewise exponential models. These models were chosen for their flexibility and convenience, as the null model is nested within each alternative. Specifically, a Weibull distribution has a two-parameter baseline hazard that allows the baseline hazard to change over time, and reduces to the exponential distribution when the scale parameter is equal to one. A Kaplan-Meier log-log survival curve plot was utilized to gauge appropriateness of the Weibull as a baseline hazard. *K*-piece exponential models allow the hazard to change at *K* distinct time points, but constrain the hazard to be constant within each interval.

The two two-piece exponential models were formulated for different purposes and were not to be directly compared. The first was hypothesis-driven; we set the cut point *τ*_1_ at 12 months to address the specific hypothesis that the hazard changes after one year of experience. The second was data-driven and estimated the optimal time of change ( ${\hat{\tau }}_{1}$) under a 2-piece exponential model, for example, to address up to what time point would a hypothetical intervention be useful. To estimate ${\hat{\tau }}_{1}$ we utilized an approach similar to one employed by Tarres et al. [18] where the cut point that yielded the largest likelihood value was chosen over all possible cut points. Bootstrap methods were used to estimate the variability of ${\hat{\tau }}_{1}$ by re-sampling with replacement 400 data sets each with 10,000 employees [24].

For computational purposes, piecewise exponential models can be rewritten as Poisson models [25], greatly simplifying estimation. Specifically, the term representing change in baseline hazard in the model can be subsumed into the vector of parameters as a dummy variable that represents change in hazard [25], making these models straightforward to fit.

### Correlation of outcomes within employee and among employees within plant

As workers contributed multiple outcomes, we applied frailty models, which included a random employee-specific multiple of the baseline hazard, to adjust for the expected correlation of responses within the same worker. We assumed the frailty terms followed a Gamma distribution. Similarly, employees from the same plant may be more similar in their outcomes than employees across plants. To address this issue we assessed the robustness of the results to a variety of models that 1) considered plant a random effect, 2) considered plant a fixed effect, with inclusion of plant-level covariates or 3) solely included plant-level covariates as fixed effects. Results were examined for heterogeneity. If results did not differ, the simplest method (3) was chosen.

### Handling of missing data

The physical demand of a particular job was a key confounder of the relationship between injury and experience. This variable was missing for a large proportion of workers; these data were previously measured by the employer for jobs at only 11 of the 207 plants. Availability of physical demand data was related to a number of plant-level and individual-level characteristics. For example, those with physical demand data were 90% male, 87% Caucasian, and at the start of a job, are approximately 43 years old and employed by the company for about 10 years. In contrast, the full cohort was 78% male, 70% Caucasian, and at the start of a job, approximately 39 years old and employed by the company for about 3.5 years. In addition a higher proportion of severe injuries are observed among those in jobs with physical demand data, where these employees are more likely to work at a union plant that performs smelting.

Excluding subjects missing at least one variable included in the model (or a complete-case (CC) analysis) would exclude 84% of the data set. Further, validity of results from a CC analysis relies on an assumption that the data are missing completely at random, which we have demonstrated is violated. In order to minimize bias in describing how the hazard of injury varies with experience, we employed multiple imputation (MI) techniques, which provide statistically valid results when the data are missing at random (MAR), a less stringent assumption about missingness that allows missingness to be correlated with observed variables only (e.g., gender) [26]. Conditional on relevant covariates (e.g., an indicator for whether the employee worked for a union plant or whether the employee worked at one of the smelting facilities), this is a reasonable assumption here. We employed the fully conditional specification approach for MI, one of the two main approaches for doing MI, described in detail by van Buuren [27]. We used *m* = 4 imputations, and as suggested by the missing data literature, confirmed the appropriateness of this number by examining stability of estimates.

### Model selection

Comparisons across models were performed using cross-validation techniques. More specifically, the data were randomly partitioned into two pieces where two-thirds of the employees are randomly selected to be in the “learning set”, while the remaining one-third serves as the “test set”, on which all models are compared. Such an approach allowed for model development such as estimation of *τ*_1_ for the optimally chosen two-piece exponential model and fair comparisons on an untouched data set. Final results, however, were presented on the entire data set. Detailed steps for model selection and fitting were provided in Appendix.

Data cleaning was performed using SAS v9.3. Model selection, model fitting and multiple imputation were performed using STATA 11.2, specifically with STATA’s ice() program [28]. All analyses were conducted after receiving approval from the Institutional Administrative Panels on Human Subjects in Medical Research (IRB).

## Results

Table 1 describes characteristics of the 81,301 employees and their corresponding 191,692 jobs. Each employee provided data on a median number of two jobs. The distribution of number of injuries was heavily skewed, as most employees experienced no injuries (mean = 0.39 and median = 0). The cohort was heavily male (78%), and largely white (70%). On average employees were 39 years old, and had been employed an average number of eight years at the company.

**Table 1:** Demographics of hourly production workers at 207 U.S. facilities of a global aluminum company employed between January 1, 1996 and December 31, 2007

	**Entire cohort**		**Physical demand cohort**	
**Cohort demographics**	**Count**	**Percent / Mean (SD)*Median***	**Count**	**Percent / Mean (SD)*Median***
***Total jobs***	191 692		33 427	
***Total unique employees***	81 301		13 427	
***Number of jobs per employee***		2.36 (2.30) *2*	3.99 (2.88) *3*	
***Number of injuries*per employee**		0.39 (0.96) *0*	0.98 (1.49) *1*	
***Gender***				
Male	63 233	77.78	12 105	90.15
Female	18 006	22.15	1 319	9.82
Unknown/Missing	72	0.09	3	0.00
***Race/Ethnicity***				
White	55 709	70.46	11 669	86.92
Black	12 371	15.65	1242	9.25
Hispanic	8340	10.55	386	2.88
Asian	1786	2.26	46	0.34
American Indian	610	0.77	66	0.49
Mixed (more than one reported)	247	0.31	16	0.12
Unknown/Missing	2238	2.75	2	0.01
***Age (years) at start of job***		39.37 (11.32) *39.23*		42.61 (10.19) *43.24*
***Tenure (years) at start of job***		8.43 (10.25) *3.62*		13.27 (11.96) *9.60*

Table 2 describes characteristics of the 31,456 relevant injuries reported by severity and type. The majority (72%) were classified as “first-aid” injuries, 15% required medical treatment, 11% resulted in restricted work, and 2% resulted in a lost workday.

**Table 2:** Characteristics of injuries in hourly production workers at 207 U.S. facilities of a global aluminum company employed between January 1, 1996 and December 31, 2007

	**Entire cohort**		**Physical demand cohort**	
**Injury characteristics**	**Count**	**Percent**	**Count**	**Percent**
***Injuries***	31 456		9549	
***Case Category***				
**First Aid**	22 748	72.32	6187	64.79
**Medical Treatment**	4598	14.62	1861	19.49
**Restricted Work**	3585	11.40	1328	13.91
**Lost Work Day**	525	1.67	173	1.81
***Injury Description***				
**Abrasion**	2591	8.24	715	7.49
**Burn**	4054	12.89	1099	11.51
**Contusion**	6983	22.20	2131	22.32
**Foreign body**	2270	7.22	625	6.55
**Fracture**	940	2.99	334	3.50
**Laceration**	5620	17.87	1427	14.94
**Acute musculoskeletal**	6283	19.97	2342	24.53
**Other**	2696	8.57	875	9.16
**Missing**	19	0.06	1	0.01

**Figure 2:**
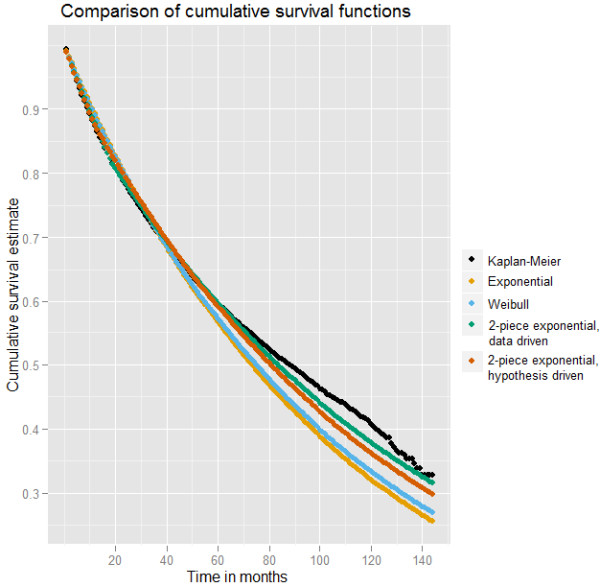
Injury free curves for job experience for test set chosen from 81,301 employees under various models.

**Figure 3:**
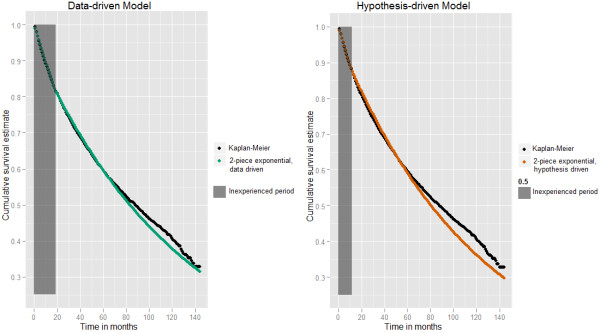
Injury free curves for job experience for test set chosen from 81,301 employees under the two-piece exponential models.

Figure 1 provides an aggregated look at the rate of injuries as a function of months on the job. The plot demonstrates decreasing injury rates (with increasing variability) over time on the job. Figure 2 compares injury free curves based on the “test” set across the four proposed models to that of the observed (estimated using the Kaplan-Meier (K-M) method). The K-M curve indicates a steep decline initially and then a slowing down in injuries. Under the null hypothesis, one expects the rate of decline to be constant over time. The curve from the exponential model, however, visually fits that of the observed least well, declining too slowly initially, and too quickly after about two years. While an improvement over the exponential model, the Weibull model appears graphically inferior to the two-piece exponential models. Note that the data-driven two-piece exponential estimated the cut point at 19 months (mean 25.32, SD 13.47). Figure 3 shows the data-driven and hypothesis-driven two-piece exponential models only, which appear largely comparable, although such a comparison is not directly relevant to our research question.

Table 3 presents results comparing the four fits from the models on the “test” set. All models considered under the alternative hypothesis prove superior to the exponential model, favoring rejection of the null that experience does not matter and that the hazard is constant over one’s time on the job. While the Weibull model proves superior to the exponential, this comparison produces the least impressive likelihood ratio test (LRT). Using graphical evidence and Bayes factors to compare each two-piece exponential to the Weibull, we find evidence in favor of the two-piece models. We did not compare the data-driven and hypothesis-driven two-piece models, as this was not a comparison of interest since the models address different hypotheses.

**Table 3:** Fit statistics and comparison of models by LRT statistic and Bayes factors using the test set

	***Exponential***	***Weibull***	***Data-driven 2-piece exponential***	***Hypothesis-driven 2-piece exponential***
**Fit statistics**				
Number of Parameters	8	9	9	9
Log-likelihood	−32576.2	−32573.6	−32447.3	−32468.2
BIC (Smaller is better)	65263.6	65272.2	65019.6	65061.5
**Model comparison**				
*LRT Statistic*				
*Bayes Factor*				
***Exponential***	NA	5.21*	257.9***	216.0***
		1.000	0.996	0.997
***Weibull***	5.21*	NA	Not nested	Not nested
	1.000		0.996	0.997
***Data-driven 2-piece exponential***	257.9***	Not nested	NA	Not nested
	1.004	1.004		1.001
***Hypothesis-driven 2-piece exponential***	216.0***	Not nested	Not nested	NA
	1.003	1.003	0.999	

Note: * denotes significance at the 0.05 level, ** denotes significance at the 0.01 level, and *** denotes significance at the 0.001 level.

Tables 4 and 5 presents the results based on fitting the two-piece exponential models. We provide both the results based on the full data set from a CC analysis, which includes 33,427 jobs on 13,427 employees, and those from an MI-based analysis, which includes 191,692 jobs on 81,301 employees. All models adjusted for the pre-specified confounders listed above. Accounting for the correlation of responses among employees within the same plant across the three methods described does not yield different estimates of the parameter of interest (results not shown). We therefore chose the simplest approach, including indicators for union and smelter status as terms in the model, which also allowed for the assessment of their impact on risk. Accounting for the correlation of responses within employee, however, yielded point estimates attenuated toward the null relative to those obtained assuming independence. A CC analysis using a frailty model and two-piece exponential baseline hazard allowing the baseline hazard to change after one year of experience estimated a 32% increase in the hazard of injury during the first year of experience (See Table 4). Similarly, a CC analysis using a frailty model for the optimally-chosen model indicated a 41% increase in hazard up to 19 months (See Table 5). The MI-based analyses yielded comparable findings, with the exception of the point estimate corresponding to whether the plant was one of the original founding plants. This makes sense, as the complete-case cohort consisted of only 11 plants with little heterogeneity in this variable. As a consequence, no association was observed between injury and being employed by an original plant in the complete-case cohort, whereas an increased risk in injury was observed in the MI-based analysis.

**Table 4:** Results from CC and MI frailty models for 2-piece exponential model with 12 month cut point (hypothesis-driven model)

	***Complete case analysis (N=33 427 jobs for 13 427 employees)***		***Multiple imputation (N=191 692 jobs for 81 301 employees)***	
	***HR (95% CI)***	***P-value***	***HR (95% CI)***	***P-value***
**0-12 Months (inexperienced period)**	1.32 (1.26, 1.38)	<.001	1.25 (1.23, 1.28)	<.001
**Male gender**	0.62 (0.57, 0.68)	<.001	0.71 (0.68, 0.73)	<.001
**Non-White**	1.08 (1.00, 1.17)	0.061	0.97 (0.93, 1.00)	0.073
**First job at company**	0.88 (0.79, 0.98)	0.017	0.90 (0.87, 0.93)	<.001
**Age at start of job**	0.98 (0.98, 0.99)	<.001	0.99 (0.98, 0.99)	<.001
**Physical demand**	1.25 (1.22, 1.29)	<.001	1.26 (1.24, 1.29)	<.001
**Smelter plant**	1.25 (1.18, 1.33)	<.001	1.30 (1.24, 1.29)	<.001
**Union plant**	1.15 (1.04, 1.27)	0.008	1.30 (1.23, 1.37)	<.001
**Original plant**	1.01 (0.83, 1.22)	0.952	1.58 (1.50, 1.65)	<.001

**Table 5:** Results from CC and MI frailty models for 2-piece exponential model with 19 month cut point (data-driven model)

	***Complete case analysis (N=33 427 jobs for 13 427 employees)***		***Multiple imputation (N=191 692 jobs for 81 301 employees)***	
	***HR (95% CI)***	***P-value***	***HR (95% CI)***	***P-value***
**0-19 Months (inexperienced period)**	1.41 (1.35, 1.47)	<.001	1.33 (1.29, 1.36)	<.001
**Male gender**	0.62 (0.57, 0.68)	<.001	0.71 (0.68, 0.73)	<.001
**Non-White**	1.08 (0.99, 1.17)	0.070	0.97 (0.93, 1.00)	0.067
**First job at company**	0.88 (0.79, 0.98)	0.017	0.90 (0.88, 0.93)	<.001
**Age at start of job**	0.98 (0.98, 0.99)	<.001	0.99 (0.98, 0.99)	<.001
**Physical demand**	1.25 (1.21, 1.29)	<.001	1.26 (1.24, 1.29)	<.001
**Smelter plant**	1.24 (1.17, 1.32)	<.001	1.30 (1.24, 1.36)	<.001
**Union plant**	1.16 (1.04, 1.28)	0.006	1.30 (1.23, 1.38)	<.001
**Original plant**	1.01 (0.83, 1.22)	0.942	1.57 (1.50, 1.65)	<.001

Our findings are consistent regarding previously identified correlates of injury risk [1,2]. Previous studies have indicated that females and Caucasians are at an increased risk of injury. As anticipated, a 1-unit increase in the scale of physical demand increased risk of injury by 25%. Similarly, those working at a smelter plant had hazards that are 1.2 times as great as those in non-smelter plants and those at union plants had hazards that are 1.2 times those at non-union plants. For every 1-year increase in age, we observed a decrease in hazard, as previously reported. Importantly, the reduction in hazard of injury by experience persisted even after adjustment for age.

A sensitivity analysis that included calendar year in the model to account for secular trends in injury during the study period did not affect inference regarding experience. A second sensitivity analysis restricting the outcome to injuries reportable to the Occupational Safety and Health Administration (OSHA) supported the conclusion that less than one year of job-specific experience yielded an increase in the hazard of injury; here, the magnitude of association was increased (HR=1.62 vs. 1.32).

## Discussion

This paper illustrates the novel use of a flexible statistical approach for evaluating the association between job experience and hazard of injury. By identifying the baseline hazard as the main parameter of interest, we were able to address the research question by comparing competing models for the baseline hazard. We found the piecewise exponential model to be particularly useful in this context with respect to goodness of fit, interpretability, as well as computational ease. Further our approach is applicable to other contexts where the baseline hazard is the main focus. After formulating our general analytic approach, we still faced a number of methodological challenges. These included correlation of observations and missing data. We addressed these issues by including a frailty term in the model to account for correlated responses among employees and through multiple imputation techniques to handle the missing data. Using our selected model, our findings demonstrated a 30% increase in the hazard of injury prior to accumulating one year of experience and even higher when only more serious, OSHA reportable injuries are included.

### Models for consideration

Because the outcome, the length of time to injury, was equivalent to the predictor of interest, experience, this precluded the use of the commonly applied Cox PH model to evaluate how experience affected hazard of injury and directed the focus to the baseline hazard. For this reason, we compared various competing parametric models for the baseline hazard. Relevant comparisons were drawn between models under the alternative (Weibull and two-piece exponential models) and the model under the null (exponential model). Favoring the more flexible model was an indication that the hazard varied by time on the job and thus, that experience mattered. Other models that we could have considered for the baseline hazard include several in the accelerated failure time family such as the log-logistic, log-normal, and generalized gamma models [29]. Other flexible approaches involving splines could have been considered as well [30]. Our approach however was to identify alternative models to the exponential that were both more flexible and that included the exponential model as a special case.

### Consideration of two different two-piece exponential models

The two two-piece exponential models were considered for slightly different purposes. The two-piece exponential that allowed the baseline hazard to change at 1 year directly addressed whether the hazard changed significantly after 1 year (an a priori specified time point of interest), suggesting that targeting an intervention prior to this period may reduce risk. The optimally-defined two-piece exponential model was fit to assess where the maximized difference in hazard under a two-piece model occurred. This provided insight into when one could be considered optimally experienced from an injury-hazard perspective, and/or up to what time point a hypothetical intervention could be considered useful. A *K*-piece exponential model with *K* > 2 may have better fit the data, as it would have allowed for even greater flexibility. Our questions were addressed, however, with these 2-piece models. Another approach would be to use cross-validation measures to optimize *K* and then estimate changes in hazard given the optimal *K*. This would answer a slightly different question, although still a potentially interesting one.

### Comparing competing models

Comparisons between the Weibull and exponential models demonstrated the Weibull model as the better fit, indicating sufficient evidence to reject the null that the hazard is constant over time. Similarly comparisons between the exponential and each two-piece demonstrated the need for such flexibility. These comparisons addressed our first question of whether the hazard was non-constant over time (i.e., whether there is a relationship between experience and injury risk). We did not formally compare the Weibull to each two-piece model using a LRT as neither model is nested within the other and thus, the behavior of the LRT statistic is not known in this case. Graphical evidence, however, suggests the superior fit of each two-piece model to the Weibull. Additionally, the likelihood values corresponding to the two-piece models were considerably larger than that of the Weibull, while the number of parameters estimated was the same. From an interpretability standpoint, the two-piece models were more appealing as they yielded parameter estimates that directly corresponded to changes in hazard at the specified time point, which may be useful in targeting interventions (while the parameter estimates for a Weibull model describe the shape of the baseline hazard, which is not directly meaningful).

### Limitations

There are some limitations to the study that merit mention. First, we have not accounted for the variable number of hours worked each month (hence opportunity for an injury) across individuals. We expect variability across employees because of business cycles, overtime and vacation schedules [4,20]. Incorporating this variability is challenging because of data limitations. Another potential concern is that we did not adjust for changes in injury risk over secular time during the study period, given that nationally and over the course of this study, injury rates have improved at the company. We have explored the implications of this by refitting our candidate models with the inclusion of time-varying indicators for calendar year. While the relationship between calendar year and the hazard of injury is as expected, after adjusting for secular trends, the influence of experience on the hazard of injury did not change (data not shown).

In addition, physical demand, an established risk factor for injury in this workforce [2], was measured in a non-representative sample of plants inducing missing data in our cohort of employees eligible for study. We utilized multiple imputation techniques to address this issue that relied on assumptions plausible for our data set.

Finally, we acknowledge that this employer and workforce may be unique; aluminum manufacturing is intrinsically more dangerous than average for the manufacturing sector while the studied employer is observably more injury-adverse than the sector average. Generalizing to manufacturing more broadly, or beyond to such domains as construction, mining or service work, while tempting, may be unjustified.

### Strengths

Although there were several limitations to our study, our goal was to illustrate the application of flexible parametric methods for studying this association in the presence of several statistical challenges. In addition our approach has application to other contexts where the focus is the baseline hazard [17-19]. As our findings are demonstrated on this large longitudinal population using rich high-quality administrative data, our analysis lends confidence that previous concerns about higher injury risk among inexperienced workers are well founded, at least in our setting. Our result is highly consistent with the prevailing literature, but is the first to characterize the reduction in hazard while managing many of the methodological issues previously ignored. Moreover, our findings are *not* confined to newly hired workers, but extend also to those longer tenured who change jobs to ones demanding new tasks. Previous studies typically equate “new” with newly-hired, whereas we considered employees as newly inexperienced for subsequent job change. As job changes are typically more frequent in the manufacturing workplace than new hires, especially in the present economic climate, we would propose that efforts to control the risks of inexperience be focused not just on new hires but on all workers “new to the job”.

## Conclusions

In summary, we have demonstrated that piecewise exponential models offer the flexibility of modeling changes in the hazard of injury with ease in interpretation. They are particularly useful in the context in which the baseline hazard is primarily of interest and where Cox PH models are inapplicable. While the Weibull model demonstrated that experience mattered, it did not provide a simple characterization of the change in hazard. In particular, the reduction at a specific time point was not described, nor was the ideal time point at which change occurred. Both of these features were easily accessible through the two formulated (hypothesis-driven and data-driven) two-piece exponential models. We encourage analysts to consider use of piecewise exponential models, which allow flexibility for changes in the hazard, are easy to fit, and provide meaningful interpretation.

## Appendix

Step 1. Randomly partition the employees into the learning (two-thirds of subjects) and test sets (remaining one-third of subjects). The *Full Cohort* contains the same respective employees.

Step 2. Estimate *τ*_1_ for the two-piece exponential model using the learning set.

Step 3: From the learning set, re-sample with replacement 400 data sets of 10,000 employees and for each, estimate *τ*_1_ to obtain an estimated variance for ${\hat{\tau }}_{1}$.

Step 4. Make relevant comparisons among proposed models on the test set using the likelihood ratio test (LRT) statistic for nested models (e.g., Weibull and exponential) and the Bayesian Information Criterion (BIC) for non-nested models (e.g., Weibull and two-piece exponential models). The two-piece models are not compared as they address different hypotheses.

Step 5. Fit the chosen model(s) with a frailty term on the *Full Cohort* performing a complete-case and MI-based analysis adjusting for pre-specified confounders.

## Abbreviations

Cox PH: Cox proportional hazards; CC: Complete case; MI: Multiple imputation; MAR: Missing at random; K-M: Kaplan-Meier; LRT: Likelihood ratio test; OSHA: Occupational Safety and Health Administration; BIC: Bayesian information criterion.

## Competing interests

Dr. Cullen serves as a senior medical advisor to Alcoa under the terms of a research contract between Stanford University and Alcoa, Inc. The other authors do not have any conflicts of interest to report.

## Authors’ contributions

All authors contributed to this work. Specifically, JK implemented all methods, helped to interpret the results, and participated in the writing of the paper. MRC posed the question, coordinated the research team and participated in all stages of the writing and editing of the paper. LC was responsible for classifying the physical demand of jobs, creating and conducting the physical demand survey, and reviewing the paper and analyses. MS oversaw the management of the data and provided feedback on analyses. BT-S assisted in the data management and data cleaning. OT oversaw the coordination between the study team and employer, particularly regarding the interpretation of findings relevant to the work environment. MD proposed the methods for addressing the research question, directed the analysis, interpreted the results, and co-wrote the manuscript. All authors read and approved the final manuscript.

## Pre-publication history

The pre-publication history for this paper can be accessed here:

http://www.biomedcentral.com/1471-2288/13/89/prepub
